# Mortality after out-of-hospital cardiac arrest in a Spanish Region

**DOI:** 10.1371/journal.pone.0175818

**Published:** 2017-04-13

**Authors:** Rosa Requena-Morales, Antonio Palazón-Bru, María Mercedes Rizo-Baeza, José Manuel Adsuar-Quesada, Vicente Francisco Gil-Guillén, Ernesto Cortés-Castell

**Affiliations:** 1 Department of Nursing, University of Alicante, San Vicente del Raspeig, Alicante, Spain; 2 Emergency Medical Service, Valencian Regional Ministry of Health, Alicante, Alicante, Spain; 3 Department of Clinical Medicine, Miguel Hernández University, San Juan de Alicante, Alicante, Spain; 4 Department of Pharmacology, Pediatrics and Organic Chemistry, Miguel Hernández University, San Juan de Alicante, Alicante, Spain; Azienda Ospedaliero Universitaria Careggi, ITALY

## Abstract

**Aims:**

To determine out-of-hospital cardiac arrest mortality in the province of Alicante (Spain) and its associated factors.

**Methods:**

Cross-sectional observational study of all patients who received cardiopulmonary resuscitation (CPR) by the Emergency Medical Services (EMS) (n = 422) in the province of Alicante in 2013. To determine associated factors, a binary logistic regression model was constructed. Primary outcome: death before arriving at the hospital. Predictive variables: gender, age, artificial respiration, prior functional status, asystole, cardiogenic aetiology, bystander CPR, time from the cardiac arrest to the arrival of the EMS and location of cardiac arrest.

**Results:**

There were 337 deaths (79.9%; 95% CI: 76.0–83.7%). Factors independently associated (p<0.05) with death were: male gender (OR = 2.11; 95% CI: 1.20–3.72; p = 0.010), asystole (OR = 1.99, 95% CI: 1.17–3.39; p = 0.012), cardiac arrest at home (OR = 2.44; 95% CI: 1.42–4.18; p = 0.001) and an increased time between arrest and EMS arrival (OR = 1.05, 95% CI: 1.01–1.09, p = 0.009). Having a worse prior functional status had a tendency towards significance (OR = 0.56, 95% CI: 0.31–1.02, p = 0.059).

**Conclusions:**

Mortality was high. The associated factors were: male gender, asystole, worse prior functional status, longer time from the cardiac arrest to the arrival of the EMS and having the cardiac arrest at home. The clearly negative impact of experiencing a cardiac arrest at home necessitates modifying training policies in Spain. These policies should be focused on providing information about CPR in schools in order to decrease the mortality of these events.

## Introduction

Out-of-hospital cardiac arrest remains an important public health problem leading to many deaths, undoubtedly due to the fact that when emergency medical services (EMS) arrive the process is already irreversible [1]. The success of emergency medical systems in this process is limited [2,3], although it has been increasing [4], possibly due to multiple factors. Some factors may be intrinsic to the patient, such as diseases or prior status, and others due to the unique circumstances of each event, including the location of the cardiac arrest, and even the particular characteristics of each EMS team [5].

The incidence of cardiac arrest is 62.3 events per 100,000 person-years in adults (34.7 in paediatrics) [6]; in Spain it is estimated at 24,500 annually, almost all of cardiac origin [1]. Nearly 95% of those affected die within minutes, receiving no cardiopulmonary resuscitation (CPR) until the arrival of specialized assistance, at which time tissue necrosis has usually already occurred [7]. Since the publication of the American Heart Association Cardiorespiratory Resuscitation Guidelines in 2005 and the European Resuscitation Council Guidelines [8,9], many communities and resuscitation systems have reported a higher level of survival in cardiac arrest victims, but even so we must take into account that more than half of these cases occur in the home [10,11]. In these cases, very few victims of cardiac arrest receive basic CPR from a family member or bystander, which is why basic CPR training in the general population could be helpful in the face of this event [12]. Indeed, this sort of training has been incorporated into the recommendations, in which importance is placed on lay persons who witness a cardiac arrest [7,8].

Due to the importance of cardiac arrest and the fact that in our setting this has not been analysed, the aim of this study was to determine its incidence in our environment, the survival rate achieved, and the factors that may be associated with a fatal outcome (age, gender, location of cardiac arrest, etc.) As a new feature, building on the work of the other Spanish authors [1], explanatory variables of death that have not been previously assessed such as functional status prior to the cardiac arrest were analysed. On the other hand, in the international setting, other authors have assessed the mortality from CPR to discharge, instead of the mortality seen by the EMS [6].

## Materials and methods

### Study setting

Alicante is a province in south-eastern Spain with 1,854,244 inhabitants in 2013 and covering an area of 5,816 square kilometres, giving a population density of 319 inhabitants per square kilometre. The healthcare system is free to access, free of direct charge and universal, as in the rest of Spain.

### Study design and participants

Persons who experienced out-of-hospital cardiac arrest and who were attended by the EMS in the province of Alicante (Spain), including traumatic arrests. The EMS in Spain include trained physicians, nurses and technicians all specialised in full emergency care (not just life support), together with all the equipment necessary to undertake advanced CPR. To contact these teams, inhabitants should use the toll-free number (#112). When a call to this number is received and it relates to a case of cardiac arrest, the coordinating doctor gives instructions to the caller over the phone about how to start resuscitation until the arrival of the EMS advanced life support team. Note that the EMS only attend cases of out-of-hospital cardiac arrest after someone has called the emergency number; thus all the cases of cardiac arrest in this study were witnessed. During the CPR, the medical team invite the family and/or witnesses to stay. Concerning education and training of the general population, multidisciplinary teams from health centres and hospitals give courses and workshops about how to act in the presence of a person with a cardiac arrest and start CPR. Finally, any initiatives for advanced directives/DNRs in those with poor functional outcome cannot be considered in out-of-hospital cardiac arrests as the relevant documents are held with the clinical history of the patient, which is not accessible from the ambulance. Thus, this should be analysed for cases of cardiac arrest that occur within a hospital or a health centre.

This cross-sectional observational study comprised all patients treated (CPR) by the EMS for out-of-hospital cardiac arrest in the province of Alicante in 2013. When a patient experiences a cardiac arrest, the EMS teams are required to register all clinical data relating to the cardiac arrest in computerized form, interviewing the witnesses present at the time of the cardiac arrest; i.e., all data are obtained in a primary manner without recourse to the patient’s medical history using the Utstein recommendations of cardiac arrest studies. This information was analysed in this study. Those patients who did not receive any type of resuscitation (due to terminal illness or excessive time from the cardiac arrest to the arrival of the EMS) were excluded. Excessive time was defined as > 8 minutes without receiving basic life support, depending on the cause of the cardiac arrest, as with effect from this time the risk of severe neurological damage is very high [7,13]. This time is increased to 15 minutes only in cases of cardiac arrest with hypothermia.

### Variables and measurements

The primary variable was death due to cardiac arrest. Secondary variables (those that could explain death) were: gender, age (years), application of assisted ventilation (endotracheal intubation or bag valve mask), prior functional status considered as a quantitative variable using the Clinical Performance Score measured by the witnesses and the EMS (0→ brain dead, comatose or persistent vegetative state; 1→ conscious with severe deficit and totally dependent; 2→ conscious and alert with moderate neurological deficit; 3→ conscious, alert and oriented with normal higher functions) [14], asystole, location of the cardiac arrest (home or other), bystander CPR, cardiogenic aetiology and time from the cardiac arrest until the arrival of the EMS. Patients were only intubated if they presented some sign of cardiac activity during the first 15 minutes of CPR and intubation was possible because the airway was accessible. Information about the variables concerning whether a basic CPR had been given, time to arrival of the EMS and the prior functional status of the patient was all obtained from the witnesses.

### Sample size calculation

Since the sample was collected without prior sample size calculation, calculation of sample size, that is, determining whether the sample used is suitable for the proposed objectives, was performed a posteriori.

Of a total of 422 patients attended by the EMS, 337 died. The aim was to estimate the incidence of mortality in our setting. To do this we assumed an expected prevalence of 84.3% [1] and a type I error of 5%. With these parameters the error in the estimation was 3.47%.

### Statistical analysis

Absolute and relative frequencies were used to describe the categorical variables, whereas means and standard deviations were applied for the quantitative variables. Crude incidence was calculated for out-of-hospital cardiac arrests attended by the EMS. A binary logistic regression model was constructed to predict our outcome (death), using all the secondary variables as independent variables. The functional status was analysed as a linear quantitative variable, because we determined that the square of this variable did not show differences with it (p = 0.335, likelihood ratio test). In other words, it is better to use this variable as a linear variable instead of power or a qualitative variable. The goodness-of-fit of the model was performed using the likelihood ratio and the Hosmer-Lemeshow test, and the area under the ROC curve (AUC) [15]. All analyses were performed with a type I error of 5%, and for each relevant parameter its associated confidence interval (CI) was calculated. All calculations were performed with IBM SPSS Statistics 19.

### Ethical issues

This study is consistent with the ethical principles of the Declaration of Helsinki and was approved by the Directorate of Emergency Services of Alicante (Regional Ministry of Universal Health Care and Public Health) in 2013, which allowed the analysis of information in a completely anonymous and encrypted form. Therefore informed consent was not required for this study.

## Results

The incidence of out-of-hospital cardiac arrest in the province in 2013 was 34 per 100,000 inhabitants, in 67% of which CPR was given by the EMS. The second most common aetiology of the arrest was respiratory disease.

**Fig 1:**
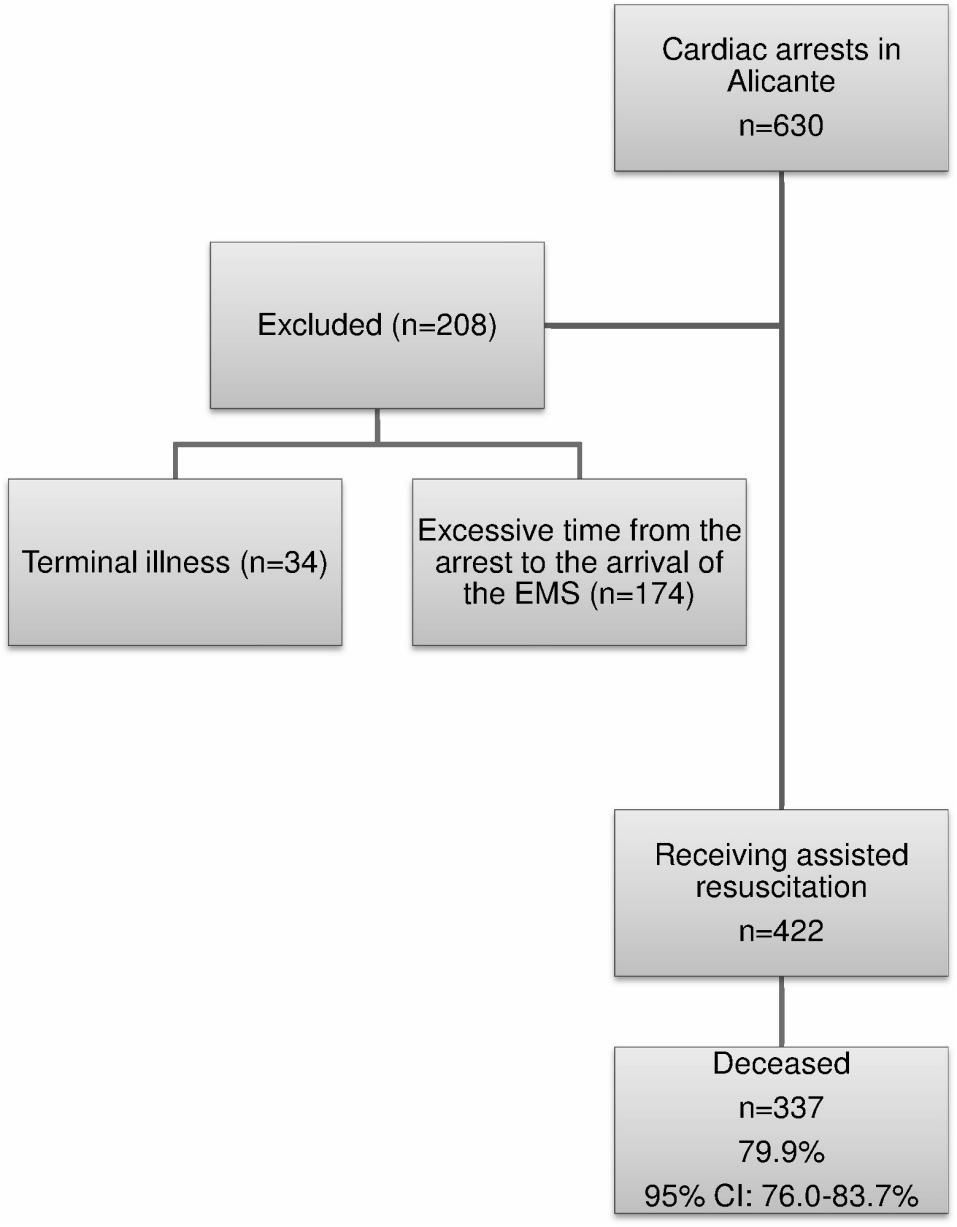
Flow diagram of the analysis of mortality in cardiac arrest attended by the Emergency Medical Services in the province of Alicante in 2013. CI, confidence interval; EMS, Emergency Medical Services.

Fig 1 shows the flow chart for the study. Of a total of 630 patients, 208 did not receive CPR by the EMS (34 due to terminal illness and 174 due to excessive time from the cardiac arrest to the arrival of the EMS). Of the 422 patients who received CPR by the EMS 337 died (79.9%, 95% CI: 76.0–83.7%).

Regarding the descriptive analysis of the sample analysed (Table 1), there was a predominance of men (70.9%), the mean age was 65.0 years, 77.7% received endotracheal intubation, the mean prior functional status was 2.8 (very close to status 3: conscious, alert and oriented with normal higher functions), 69.4% presented asystole, the predominant location of the cardiac arrest was the home (55.5%) and the mean time from the cardiac arrest until the arrival of the EMS was 14.1 min.

**Table 1:** Analysis of mortality in cardiac arrest attended by the Emergency Medical Services in the province of Alicante in 2013.

	Total	Mortality	Adjusted OR	p-value
	n = 422	n = 337(79.9)	(95% CI)	
Variable	n(%)/x±s	n(%)/x±s		
Gender:				
Male	299(70.9)	245(72.7)	2.11(1.20–3.72)	0.010
Female	123(29.1)	92(27.3)	1	
Age (years)	65.0±16.3	64.8±15.8	1.00(0.98–1.01)	0.752
Endotracheal intubation:				
Yes	328(77.7)	253(75.1)	0.62(0.28–1.34)	0.222
No	94(22.3)	84(24.9)	1	
Functional status (prior)	2.8±0.6	2.7±0.7	0.56(0.31–1.02)	0.059
Asystole:				
Yes	293(69.4)	248(73.6)	1.99(1.17–3.39)	0.012
No	129(30.6)	89(26.4)	1	
Location of cardiac arrest:				
Home	234(55.5)	201(59.6)	2.44(1.42–4.18)	0.001
Others	188(44.5)	136(40.4)	1	
Bystander cardiopulmonary resuscitation:				
Yes	263(62.3)	214(63.5)	1.30(0.76–2.25)	0.340
No	159(37.7)	123(36.5)	1	
Cardiogenic aetiology:				
Yes	319(75.6)	248(73.6)	0.60(0.30–1.18)	0.138
No	103(24.4)	89(26.4)	1	
Time (min)^a^	14.1±9.1	14.9±9.5	1.05(1.01–1.09)	0.009

Abbreviations: n(%), absolute frequency (relative frequency); x±s, mean ± standard deviation; OR, odds ratio; CI, confidence interval. Prior functional status is defined as: 0 = Brain dead, comatose or persistent vegetative state; 1 = Conscious with severe deficit. Totally dependent; 2 = Conscious and alert with moderate neurological deficit; 3 = Conscious, alert and oriented with normal higher functions. Goodness of fit of the multivariate model: χ^2^ = 49.0 p<0.001.

Hosmer-Lemeshow test: p = 0.054.

^*a*^, time from the arrest until the arrival of the Emergency Medical Service.

**Fig 2:**
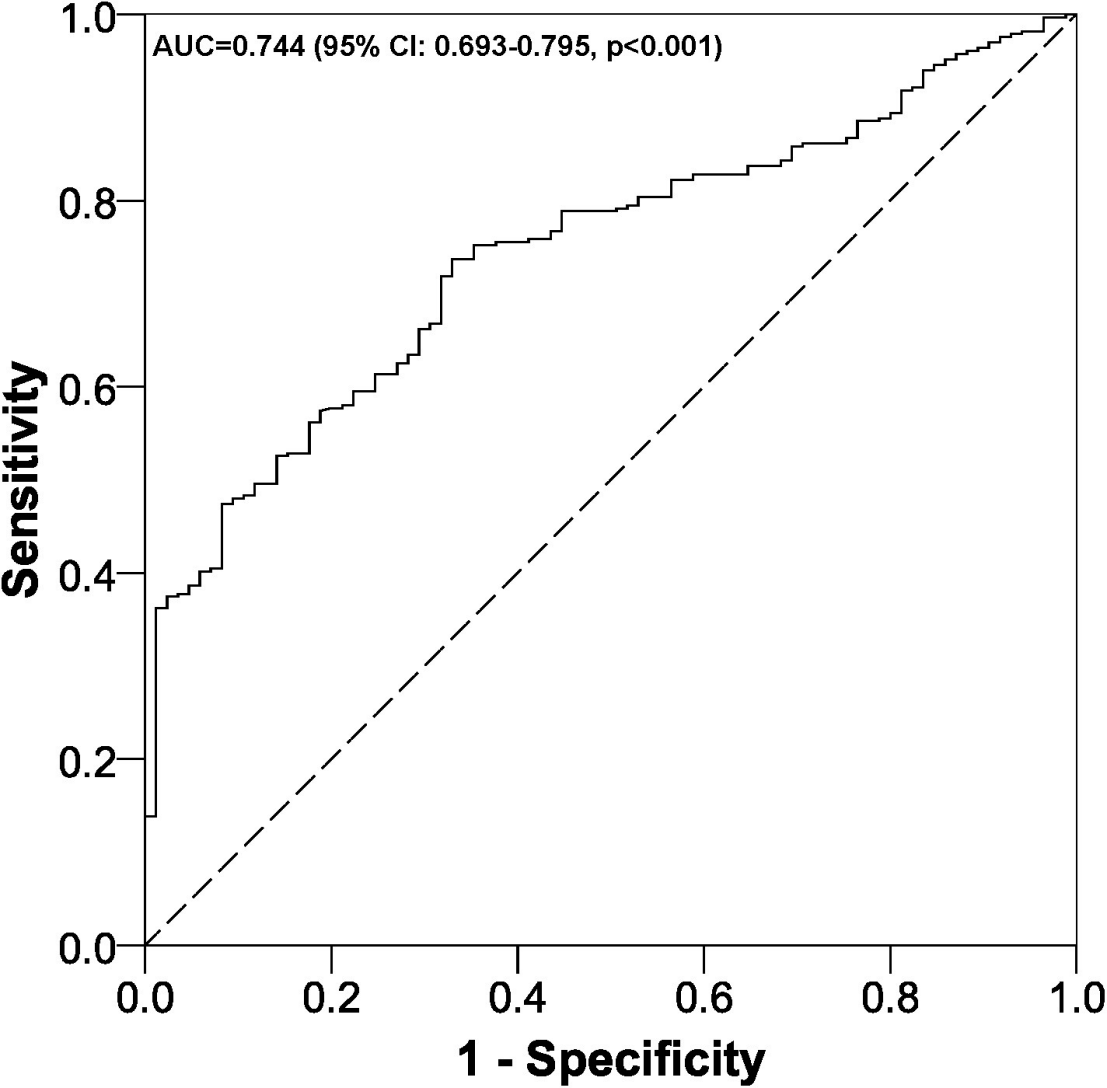
ROC curve multivariate logistic regression model. AUC, area under the ROC curve; CI, confidence interval.

The factors significantly (p<0.05) associated with death after out-of-hospital cardiac arrest were (Table 1): male gender, having asystole, the event taking place in the patient's home and increased time from the cardiac arrest until the arrival of the EMS. Moreover, having a worse functional status had a tendency towards significance (p = 0.059). The multivariate model was significant (p<0.001) and showed an AUC of 0.744 (Fig 2). The p-value for the Hosmer-Lemeshow test was 0.054.

## Discussion

### Summary

Our study found that four out of five patients in whom CPR was attempted died. Factors independently associated with mortality included male gender, asystole, longer time between the cardiac arrest and the arrival of the EMS, and experiencing the cardiac arrest at home. Additionally, having a worse functional status prior to the cardiac arrest was almost significant (p = 0.059>0.05, not significant). The factors analysed together satisfactorily explained death, as an AUC above 74% was found.

### Strengths and limitations of the study

The main strength of this study is the novel assessment of the influence of functional status prior to the cardiac arrest, asystole and the type of resuscitation used, examining the difference between performing endotracheal intubation and bag valve mask ventilation. Additionally, the sample size of our study had a statistical power close to 100%.

With regard to limitations, to minimize selection bias, all out-of-hospital cardiac arrests in our area (which are always recorded by the EMS teams) over a specified period of time were selected. Concerning information bias (possible errors when we were measuring our variables), those variables that are always collected by the same teams were evaluated, thus minimizing this type of error. Finally, other variables that could influence mortality, such as the quality of the CPR provided, pulseless electrical activity, ventricular fibrillation and pulseless ventricular tachycardia [16,17], were not assessed. However, the variables selected explained death satisfactorily, with an AUC of 74%.

### Comparison with the existing literature

First, the prevalence of the out-of-hospital cardiac arrests (34 per 100,000 person-years) was in the range of the international setting (range between 19.2 and 150.1) [6]. Second, the prevalence of death in the patients who had cardiac arrests and received CPR was nearly 80%. If we compare this value with the values reported by other authors, we note that our prevalence is very similar [1]. Concerning the associated factors, we found that male gender, having asystole, a longer time from the cardiac arrest until the arrival of the EMS and the cardiac arrest occurring in the patient's home were significantly associated with increased mortality. There is controversy regarding gender, and it seems that this association may vary by age groups [18].

The work by the Basque Country team found several factors associated with out-of-hospital death [1], including a clinical condition of asystole, an age greater than or equal to 65 years, a time to resuscitation equal to or greater than eight minutes and the cardiac arrest occurring at home. Of the factors studied by both the Basque group and our group, there is agreement regarding asystole, the increased time to arrival of the EMS, and the place of the cardiac arrest. In our study there were no differences in mortality according to age. We believe this could be due to our having analysed this variable quantitatively whereas other authors used age groups (above and below 65 years of age) [1,19]. On the other hand, and very close to significance (p = 0.059), was the association between a worse prior functional status and increased mortality. This variable has not been analysed by other authors. This relationship was logical and expected, because an older age was associated with a worse functional status. The functional status of patients who are hospitalized after a cardiac arrest has been assessed, with higher mortality found in those in an unconscious state and functional class IV [20]. Regarding the type of assisted ventilation, a variable analysed by our group that has not been assessed by others, we found no significant differences (p = 0.222) between mortality and whether the patient was intubated or not. Nonetheless, as seen in Table 1, the difference in mortality rates between the two techniques was 12% which, whilst non-significant, seems to be in line with previous reports [21]. This is consistent with the latest recommendations, which advocate chest compressions followed by ventilation and endotracheal intubation [7,13].

We stress that many studies have assessed mortality from cardiac arrest, as well as its associated factors, once the patient reaches the hospital after being resuscitated. In these patients, about 90% will eventually die or have serious neurological sequelae [22], which indicates that we have a long road ahead with respect to improving survival [23].

### Implications for practice and research

A recent phenomenon in emergency and critical care is the presence of family members during resuscitation events. It remains controversial in most institutions, but there is increasing evidence that the experience has positive effects for family members [12]. Therefore, as a future study, we intend to determine whether the training of family members improves their confidence and impact on any future crisis. In some countries, CPR and first aid courses are offered to the community through Community Centre CPR training [24], but this is not the case in Spain. Similarly, the distribution of defibrillators in public places has been shown to be effective, based on their location [25], and this practice has recently been implanted in Spain, though not the training of the lay public in their use or possible legal cover thereof [22].

The results of this study show the urgent need to establish CPR training courses for the general Spanish population, with the ideal setting for this being the school during childhood. As with other health-related subjects, like sexuality and alimentation, health professionals specialised in CPR should attend schools and colleges to train the students in this important subject. The consequence of this should be a reduction in mortality as basic CPR considerably increases the likelihood of survival.

Studies including the distance from the place of the cardiac arrest to the point of origin of the EMS teams are needed as this could be an important factor when assessing mortality [22], and even a telephone advice service for those present at a cardiac arrest until the arrival of the EMS [26].

The need for further research to better predict the outcome of CPR is indicated. This increased knowledge can affect a higher quality of CPR practiced either by professionals or bystanders witnessing the cardiac arrest, thus improving survival [16,17].

## Conclusions

Cardiac arrest is an event that very often leads to mortality. The factors associated with out-of-hospital death in our study were: male gender, asystole, increased time from the cardiac arrest to the arrival of the EMS, worse prior functional status, and the event occurring at home. The clear negative impact of the cardiac arrest occurring at home should lead to modification of training policies in Spain.

## Supporting information

S1 Dataset Database of our study. (XLSX)
